# Development and validation of mass reduction prediction model during torrefaction using biomass chemical composition analysis

**DOI:** 10.1371/journal.pone.0323940

**Published:** 2025-05-23

**Authors:** Sunyong Park, Kyeong Sik Kang, Kwang Cheol Oh, Seok Jun Kim, Paudel Padam Prasad, Seon Yeop Kim, Ha Eun Kim, Jae Youl Shin, DaeHyun Kim

**Affiliations:** 1 Agriculture and Life Science Research Institute, Kangwon National University, Chuncheon-si, Republic of Korea; 2 Department of Interdisciplinary Program in Smart Agriculture, Kangwon National University, Chuncheon-si, Republic of Korea; 3 Department of Biosystems Engineering, Kangwon National University, Chuncheon-si, Republic of Korea; University of Oklahoma, UNITED STATES OF AMERICA

## Abstract

Thermochemical processes employ heat to transform biomass into energy. In these processes, heat supply and biomass type can affect the products, therefore understanding them is critical. Confirming these changes directly requires time and resources. Several hypotheses have been proposed to explain these changes. So, the purpose of this work was to investigate mass loss during thermochemical reactions utilising available kinetic parameters. This study comprised previously pyrolysed herbal agricultural biomass (soybean pod, corncob), woody agricultural biomass (pepper stem, grape pruning branch), and forestry biomass (wood pellet, bamboo). Temperature fluctuations were studied using a 1D temperature prediction model and evaluated using kinetic parameters. The findings anticipated using prior research’ kinetic parameters differed by up to 20% from the experimental results. As a result, some of the kinetic parameters were adjusted. The prediction model with the changed parameters outperformed the prior results, with an RMSE of 2.0607 for wood pellets and 5.9754 for soybean pods. The results obtained using grape pruning branches, bamboo, and corncobs confirmed the mass reduction predicted by prior studies. This study revealed the capacity to estimate mass loss without using thermogravimetric measurements, and future predictions should include a broader spectrum of biomass materials.

## Introduction

Current research aims to transform biomass into different forms using thermochemical techniques for practical applications. Thermochemical processes entail subjecting biomass to elevate temperatures, either in the presence or absence of oxygen. The processes are categorised as low-temperature processes, namely torrefaction, pyrolysis, gasification, and combustion. Especially, interest in torrefaction and pyrolysis has grown due to the production of alternative solid fossil fuel replacements. So, several studies have investigated mass loss patterns during biomass torrefaction and pyrolysis. Gajera et al. (2022) analyzed the thermal behavior of wheat straw and groundnut stalk and observed a direct correlation between temperature increase and mass reduction [1]. Similarly, Tsai et al. (2020) examined caltrop husk torrefaction and identified a significant decline in mass at temperatures between 250°C and 300°C [2]. In another study, Helwani et al. (2020) explored the torrefaction of empty fruit bunches and reported that mass loss is closely related to energy yield, suggesting its critical role in optimizing biomass-to-energy conversion processes [3]. Kim et al. (2022) conducted torrefaction process for improving fuel characteristic of kneaf, achieving 73.8–96.6% mass yield [4].

Abdullah and Wu (2009) investigated properies and grindability of biochar as a soild biofuel [5]. Park et al. (2023c) produced biochar using unused agro-byproduct and evaluated as soild biofuel and soil amendment [6].

But the targeted process yield in these thermochemical processes might vary considerably based on the specific biomass and process conditions. In order to tackle this issue, several models have been suggested for process prediction due to the time and resource constraints associated with conducting comprehensive trials.

**Fig 1:**
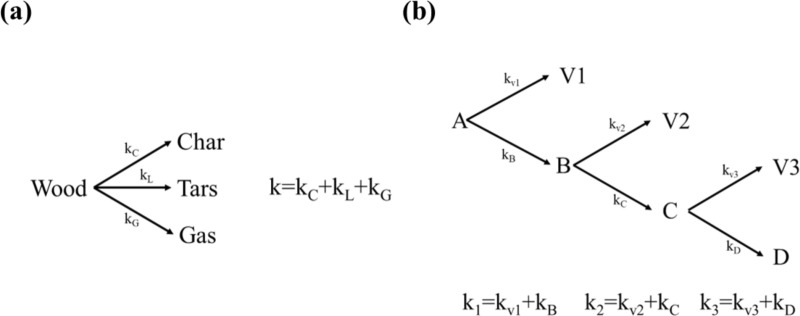
Summary of (a) one-component (b) semi-global reaction mechanism k is kinetic parameter, V stands for volatile matters and B and C refer intermediate matter.

Several mechanisms have been proposed to explain the pyrolysis and combustion of biomass. The single-component mechanism. Shafizadeh & Chin (1977) posits that biomass breakdown is a single first-order reaction (Fig 1) [7], simplifying the study but failing to grasp the complexities of biomass pyrolysis. On the other hand, the semi-global reaction mechanism [8–12] includes numerous reactions for cellulose, hemicellulose, and lignin, providing a more detailed picture of the process. However, the increased complexity necessitates extra kinetic parameters, which are frequently determined using thermogravimetric analysis (TGA). While these models offer useful insights, they confront a number of problems. The majority of existing models are based on TGA data, which does not accurately depict large-scale torrefaction or combustion settings due to discrepancies in heat and mass transfer dynamics. Furthermore, the kinetic parameters acquired from TGA tests are unique to the tested biomass and experimental setup, limiting their generalisability.

Previous work has also suggested predictive models that rely on the decrease in alterations in cellulose, hemicellulose, and lignin. [13–16]. Prior research has investigated the properties of biomass, the process of material release, and the chemical transformations occurring in the gas phase. These findings have been confirmed. The pyrolysis process was validated as a model [17–21]. However, previous models have limitations in predicting long-term mass reduction due to their reliance on short-duration experiments. Additionally, kinetic parameters used in previous studies show variability based on experimental conditions, making generalization difficult.

To address these gaps, this study aims to develop a mass reduction prediction model based on refined kinetic parameters, enabling more accurate predictions without the need for extensive experimental validation. Instead of relying on the short-term results from previous research, the objective is to analyse and study the actual thermochemical changes that take place during a process lasting for up to one hour. Pyrolysis processes should be able to more easily anticipate the quantity of mass that is created over an extended period of time thanks to this improvement.

## Materials and method

### Materials

This study included a total of six distinct categories of biomass, encompassing two varieties of herbal biomass, two varieties of woody biomass, as well as wood pellets and bamboo. The herbal agricultural biomass utilised in this study consisted of soybean pod (BE) and maize cob (CC), which were procured from Hanjung SS Co., Ltd. Both types of biomass were present in the form of feed pellets. The study utilised pepper stem (PP) as forms of woody agricultural biomass. The samples of PP were obtained from agricultural sites located in Gochang-gun, Chungcheongnam-do, Korea, and Paju-si, Gyeonggi-do, Korea. Collected PP was subsequently pelletized using a pelletizer manufactured by Geumgang ENG in Korea, namely the SP-75 model. The wood pellets (WP) used in this study were sourced from the Yeoju Forestry Association located in Yeoju-si, Korea. Bamboo specimens, aged three years or older, were acquired from Korea Bamboo Co. Ltd., located in Hapcheo-gun, Gyeongsangnam-do, Korea. The results of various research experiments were presented based on previous research [22].

### Numerical simulation

**Fig 2:**
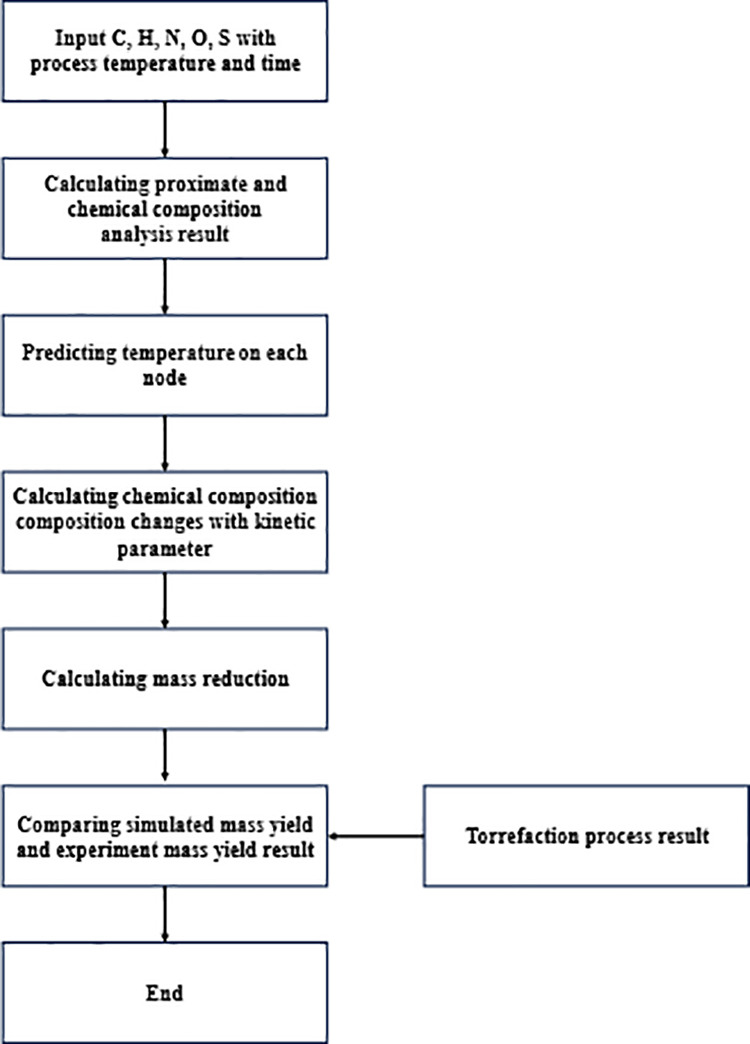
Flowchart of this study.

The mass reduction prediction model developed in this study consists of two integrated components: (1) a one-dimensional temperature prediction model and (2) a chemical composition decomposition kinetics model. The temperature model estimates biomass temperature profiles during torrefaction, while the decomposition kinetics model predicts the breakdown of cellulose, hemicellulose, and lignin. This can be readily depicted using a flow chart (Fig 2).

### One-dimensional temperature prediction model

F The model utilised in this study was based on the model proposed by Oh et al. [11]. First, enter the elemental analysis results of the untreated biomass. Then, proximate analysis and chemical composition are predicted according to the formula of previous research [23,24]. The torrefaction process had been conducted using a batch-type reactor [25,26]. The simulation was performed using Matlab (R2020b, Mathworks, MA, USA). The Matlab code utilised in this investigation is condensed in S1 File.

In this scenario, a direct numerical method was used to forecast variations in temperature within the biomass during the process of temporary heat conduction. The biomass was partitioned into several segments, with each segment including imaginary nodes located at their respective centres [27–30]. The biomass was partitioned into 55 segments, with a virtual node positioned in the centre of each segment. A limited control volume method was employed to conduct an energy balancing. In Eq (1), the left-hand side reflects the rate of change in energy content of the nodes. The first and second terms on the right-hand side correspond to the rates of heat conduction on the right and left surfaces, respectively [31,32].


$$\rho A_{b}\Delta xC_{p}\frac{dT_{m}}{dt}\;=kA_{b}\frac{T_{m+1}-T_{m}}{\Delta x}+kA_{b}\frac{T_{m-1}-T_{m}}{\Delta x}\;$$ (1)

The subscript “m” denotes spatial variation, “A_b_” represents the boundary area, “Δx” is the distance between nodes, “r” stands for the density of the biomass, and “C_P_” and “k” correspond to the specific heat and heat transfer coefficients of biomass, respectively. Woody biomass and herbaceous biomass have different thermal properties, so their respective specific heat capacities (Cp) were used. These values were determined by Eqs. (2 and 3) [33–37]. Thermal conductivities were used from previous studies (Eq. (4)) [35,36,38], respectively.


$$C_{p}=1112+4.85(T-273.15)$$ (2)

$$C_{p}=1.3294T+674.3$$ (3)

$$k_{w}=0.13+3\times 10^{-4}(T-273.15)$$ (4)

### Chemical composition decomposition kinetic model

**Fig 3:**
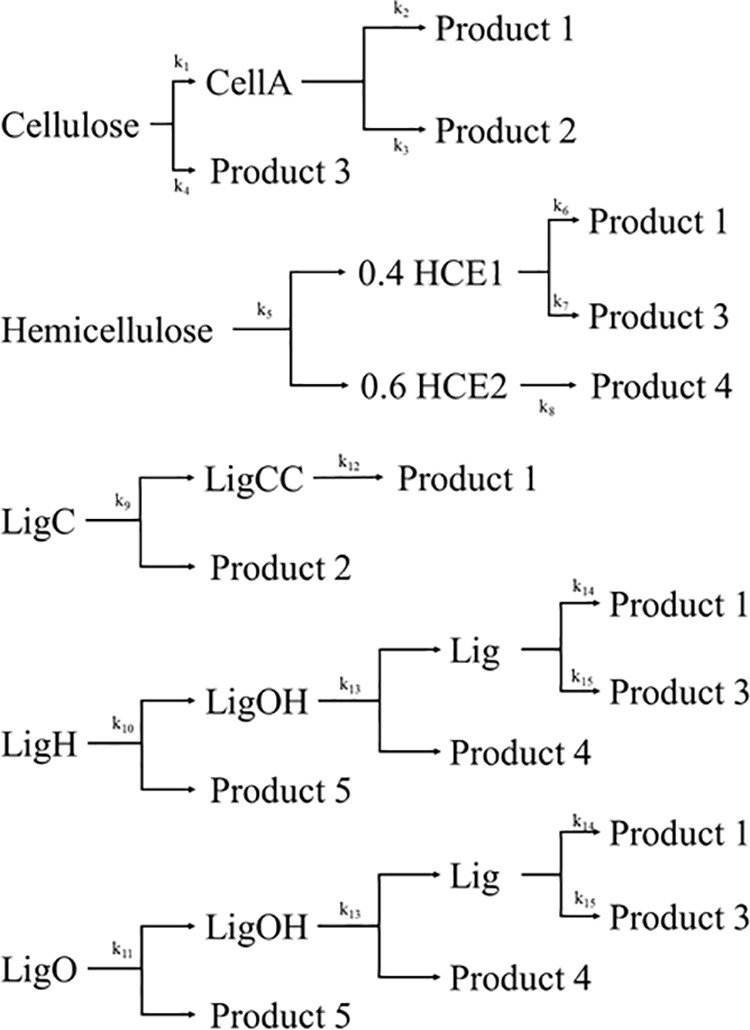
Schematic of mass reduction model.

In order to accurately simulate the thermal degradation of biomass particles, it is essential to have a comprehensive understanding of the biomass composition. The reason for this is that biomass consists of a diverse range of components that undergo unique pyrolysis processes. Biomass mostly consists of cellulose, hemicellulose, and lignin. Additionally, there could exist minor constituents such as extractive species, protein, and ash. However, acquiring accurate information regarding the biochemical composition, which would reveal the exact quantities of these constituents, is not easily attainable due to persistent difficulties and ambiguities linked to existing measuring techniques. In order to tackle this problem, earlier studies utilised a method that indirectly determined the chemical makeup of biomass by analysing its elemental composition [23]. The pyrolysis kinetics of biomass are represented by a simplified lumped kinetic model, which is derived from the more intricate kinetics of cellulose, hemicellulose, and lignin degradation seen in previous studies [39–41]. The schematic was figured out as Fig 3. These reactions and their associated kinetic parameters have been continually refined and expanded as new experimental data became available [42]. The assumption made here is that each reference component undergoes decomposition independently through first-order reactions. This kinetic model comprises 20 solid species, 17 volatiles, and 5 gas species, constituting a total of 24 reactions. Tables 1 and 2 list the solid, volatile, and gaseous species, respectively, while Table 3 provides a summary of the kinetic parameters. Moisture content (MC) of woody biomass and bamboo was set at 12%, and herbaceous biomass was set at 15%. In previous studies, split factors were used to divide LigC, LigH, and LigO based on the plot method [17,18,43,16]. However, in this study, LigC, LigH, and LigO were averaged based on the results of a previous study [42], and the results were shown in Table 4.

**Table 1:** Solid species and trapped gases in the simulation.

Name	Note	Formula
CELL	Cellulose	C_6_H_10_O_5_
CELLA	Activated Cellulose	C_6_H_10_O_5_
HECELL	Hemicellulose	C_5_H_8_O_4_
HCE1	Activated hemicellulose 1	C_5_H_8_O_4_
HCE2	Activated hemicellulose 2	C_5_H_8_O_4_
LIGC	Carbon-rich lignin	C_15_H_14_O_4_
LIGH	Hydrogen-rich lignin	C_22_H_28_O_9_
LIGO	Oxygen-rich lignin	C_20_H_22_O_10_
LIGCC	Carbon-rich lignin 2	C_15_H_14_O_4_
LIGOH	OH-rich lignin	C_19_H_22_O_8_
LIG	Lignin	C_11_H_12_O_4_
Char	Char	C
G_CH4	Trapped CH_4_	CH_4_
G_H2	Trapped H_2_	CO
G_CO	Trapped CO	CO
G_CO2	Trapped CO_2_	CO_2_
G_COH2	Trapped COH_2_	COH_2_
G_C2H4	Trapped C_2_H_4_	C_2_H_4_
G_CH3OH	Trapped CH_3_OH	CH_3_OH
Ash	Ash	Ash

**Table 2:** Volatile and gasous species in the simulation.

Name	Note	Formula
**Volatile**		
HAA	Hydroxyacetaldehyde	C_2_H_4_O_2_
GLYOX	Glyxoal	C_2_H_2_O_2_
CH3CHO	Acetaldehyde	CH_3_CHO
HMFU	5-hydroxymethyl-furfural	C_6_H_6_O_3_
C2H5CHO	Propionaldehyde	C_2_H_5_CHO
CH3OH	Methanol	CH_3_OH
CH2O	Formaldehyde	CH_2_O
H2O	Water	H_2_O
HCOOH	Formic acid	HCOOH
C3H6O2	3-hydroxypropanal	C_3_H_6_O_2_
C5H4O2	Furfural	C_5_H_4_O_2_
EtOH	Ethanol	C_2_H_5_OH
CH3COOH	Acetic acid	CH_3_COOH
C9H10O2	p-Coumaryl alcohol	C_9_H_10_O_2_
C6H5OH	Phenol	C6H5OH
C11H12O4	Acylaldehyde	C_11_H_12_O_4_
LEVO	Levoglucosan	C_6_H_10_O_5_
**Gases (Non-condensable)**		
CO	Carbon monoxide	CO
CO2	Carbon dioxide	CO_2_
H2	Hydrogen	H_2_
C2H4	Ethylene	C_2_H_4_
CH4	Methane	CH_4_

**Table 3:** Multiple-Step Kinetic Model for Biomass [18,42].

Pyrolysis Reactions				Kinetic Parameters A(s^-1^), Ea(kcal/kmol)
**Cellulose**				
1	Cell	→	CellA	1.5 × 10^14^ × exp(-47000/RT)
2	CELLA	→	0.4C2H4O2 + 0.05C2H2O2 + 0.15CH3CHO + 0.2C6H6O3 + 0.35C2H5CHO + 0.15CH3OH + 0.3CH2O + 0.61CO + 0.36CO2 + 0.05H2 + 0.93H2O + 0.02HCOOH + 0.05C3H6O2 + 0.05G_CH4 + 0.2G_H2 + 0.61Char	2.5 × 10^6^ × exp(-19100/RT)
3	CELLA	→	C6H10O5	3.3 × T × exp(-10000/RT)
4	CELL	→	5H2O + 6CHAR	6 × 10^7^ × exp(-31000/RT)
	**Hemicellulose**			
5	HECELL	→	0.58HCE1+0.42HCE2	1 × 10^10^ × exp(-31000/RT)
6	HCE1	→	0.6C5H8O4 + 0.2C3H6O2 + 0.12C2H2O2 + 0.2C5H4O2 + 0.4H2O + 0.08G_H2 + 0.16CO + 0.4H2O + 0.79CO2 + 0.05HCOOH + 0.69CO + 0.01G_CO + 0.01G_CO2 + 0.35G_H2 + 0.3CH2O + 0.9G_COH2 + 0.625G_CH4 + 0.375G_C2H4 + 0.875Char	3 × T × exp(-11000/RT)
7	HCE1	→	0.4H2O + 0.79CO2 + 0.05HCOOH + 0.69CO + 0.01G{CO} + 0.01G{CO2} +0.35G{H2} + 0.3CH2O + 0.9 G{COH2} + 0.625 G{CH4} + 0.375 G{C2H4} + 0.875 CHAR	1.8 × 10^–3^ × T × exp(-3000/RT)
8	HCE2	→	0.2H2O + 0.275CO + 0.175CO2 + 0.4CH2O + 0.1C2H5OH + 0.05C2H4O2 + 0.35CH3COOH + 0.025HCOOH + 0.25G_CH4 + 0.3G_CH3OH + 0.225G_C2H4 + 0.4G_CO2 + 0.725G_COH2 + Char	5 × 10^9^ × exp(-31500/RT)
	**Lignin**			
9	LIGC	→	0.35LIGCC + 0.1C9H10O2 + 0.08C6H5OH + 0.41C2H4 + H2O + 0.7G_COH2 + 0.3CH2O+0.32CO + 0.495G_CH4 + 5.735Char	1 × 10^11^ × exp(-37200/RT)
10	LIGH	→	LIGOH + 0.5C2H5CHO + 0.5C2H4 + 0.2C2H4O2 + 0.1CO + 0.1G_H2	6.7 × 10^12^ × exp(-37500/RT)
11	LIGO	→	LIGOH + CO2	3.3 × 10^8^ × exp(-25500/RT)
12	LIGCC	→	0.3C9H10O2 + 0.2C6H5OH + 0.35C2H4O2 + 0.7H2O + 0.65CH4 + 0.6C2H4 + H2 + 1.4CO + 0.4G_CO + 6.75Char	1 × 10^4^ × exp(-24800/RT)
13	LIGOH	→	LIG + 0.9H2O + 0.1CH4 + 0.6CH3OH + 0.1G_H2 + 0.3 G_CH3OH + 0.05CO2 + 0.55CO + 0.6G_CO + 0.05HCOOH + 0.85G_COH2 + 0.35G_CH4 + 0.2G_C2H4 + 4.15Char	1 × 10^8^ × exp(-30000/RT)
14	LIG	→	0.7C11H12O4 + 0.3C6H5OCH3 + 0.3CO + 0.3G_CO + 0.3CH3CHO	4 × T × exp(-12000/RT)
15	LIG	→	0.6H2O + 0.4CO + 0.2CH4 + 0.4CH2O + 0.2G_CO + 0.4G_CH4 + 0.5G_C2H4 + 0.4G_CH3OH + 2G_COH2 + 6Char	8.3 × 10^–2^ × T × exp(-8000/RT)
16	LIG	→	0.6H2O + 2.6CO + 1.1CH4 + 0.4CH2O + C2H4 + 0.4CH3OH + 4.5Char	1 × 10^7^ × exp(-24300/RT)
	**Metaplastic**			
17	G_CO2	→	CO2	1 × 10^6^ × exp(-24000/RT)
18	G_CO	→	CO	5 × 10^12^ × exp(-50000/RT)
19	G_COH2	→	CO + H2	1.5 × 10^12^ × exp(-71000/RT)
20	G_H2	→	H2	5 × 10^11^ × exp(-75000/RT)
21	G_CH4	→	CH4	5 × 10^12^ × exp(-71500/RT)
22	G_CH3OH	→	CH3OH	2 × 10^12^ × exp(-50000/RT)
23	G_C2H4	→	C2H4	5 × 10^12^ × exp(-71500/RT)
	**H**_**2**_ **O Evaporation**			
24	MC	→	H2O	1 × T × exp(-8000/RT)

**Table 4:** Distributions of LigC, LigO, LigH depending on the type of biomass.

	LigC	LigO	LigH
**Hardwood**	0.170	0.510	0.320
**Softwood**	0.342	0.569	0.089
**Grass**	0.376	0.394	0.229

Thus, it proved that different types of biomass underwent decomposition. During cellulose decomposition, the only remaining solids are cellulose and CellA, which have not undergone decomposition. Therefore, prognostications were formulated on the basis of: According to Eq (5), cellulose undergoes conversion to produce CellA or other substances. CellA undergoes decomposition into several compounds via reactions k2 and k3. The equation utilised was as follows:


$$Cell_{f}=Cell_{i}-\sum \frac{dCell}{dt}=Cell_{i}-\sum k_{1}Cell_{t}-\sum k_{4}Cell_{t}$$ (5)

$$CellA_{f}=\sum \frac{dCellA}{dt}=k1Cell_{t}-k_{2}CellA_{t}-k_{3}CellA_{t}$$ (6)

k_x_ stand for xth kinetic parameters. In subscription, t refers time, f and i stand for final and initial, respectively.

In the case of hemicellulose, hemicellulose is decomposed into HCE1 and HCE2 through k5 (Eq.7). For HCE1, it is decomposed through k_6_ and k_7_ (Eq. 8), and for HCE2, it is decomposed through k_8_ (Eq. 9).


$$Hemi_{f}=Hell_{i}-\sum \frac{dHemi}{dt}=Hell_{i}-\sum k_{5}Hemi_{t}$$ (7)

$$HCE1=\sum \frac{dHCE1}{dt}=\sum k_{5}Hemi_{t}-\sum k_{6}HCE1_{t}-\sum k_{7}HCE1_{t}$$ (8)

$$HCE2=\sum \frac{dHCE2}{dt}={\sum k}_{5}Hemi_{t}-\sum k_{8}HCE2_{t}$$ (9)

Regarding lignin, LigC, which contains a significant amount of carbon, exhibits only a single alteration, similar to cellulose and hemicellulose. Nevertheless, LigH and LigO, which contain a significant amount of hydrogen or oxygen, undergo decomposition to generate LigOH, and LigOH further decomposes to provide Lig. The changes were represented as equations 10–14.


$$LigC_{f}=LigC_{i}-\sum \frac{dLigC}{dt}=LigC_{i}-\sum k_{9}LigC_{t}\;$$ (10)

$$LigCC_{f}=\sum \frac{dLigCC}{dt}=\sum k_{9}LigC_{t}-\sum k_{12}LigCC_{t}$$ (11)

$$LigH_{f}=LigH_{i}-\sum \frac{dLigH}{dt}=LigH_{i}-\sum k_{10}LigH$$ (12)

$$LigO_{f}=LigO_{i}-\sum \frac{dLigO}{dt}=LigO_{i}-k_{11}LigO_{t}$$ (13)

$$LigOH_{f}=\sum \frac{dLigOH}{dt}=\sum k_{10}LigH_{t}+\sum k_{11}LigO_{t}-\sum k_{13}LigOH_{t}-\sum k_{14}Lig_{t}-\sum k_{15}Lig_{t}$$ (14)

The total mass was calculated as follows (Eq. 15).


$$Mass=\sum_{t=1}^{T_{f}}\sum_{i=1}^{node}Cell+CellA+Hemi+HCE1+HCE2+LigC+LigCC+LigH+LigO+LigOH+Captures+Ash$$ (15)

where, captures refers metaplastic such as G_CO2 and G_CO

## Results and discussion

### Simulation with previous kinetic parameters

The simulation utilised kinetic parameters derived from previous studies [18,42], The results were compared to the findings of the WP experiment, as shown in Table 5. The simulated mass yield varied between 88.06% and 68.26%, but the experimental findings ranged from 91.36% to 72.52%. The simulation findings indicated that the cellulose content ranged from 38.26% to 41.00%, hemicellulose content varied from 2.34% to 20.50%, and lignin content ranged from 33.67% to 56.40%. The errors ranged from 0.01 to 16.23%p for cellulose, 1.26 to 16.44%p for hemicellulose, and 1.82 to 9.12%p for lignin. Specifically, the most significant discrepancy was found in the case of cellulose, which was ascribed to the simulated degradation mass yield being slower than the real degradation mass yield of cellulose. The root mean square error (RMSE) ranged from 1.7927% to 3.0029%, and the coefficient of determination (R^2^) ranged from 0.4724 to 0.8803. In particular, hemicellulose exhibited a lower level of accuracy. This seemed to be because hemicellulose is composed of various types of 5-carbon sugars (xylose, arabinose) and 6-carbon sugars (mannose, galactose), and the difference in thermal decomposition occurred because the composition of the sugars constituting each hemicellulose was different.

**Table 5:** Simulation result with previous kinetic parameters of WP.

		Mass			Cellulose			Hemicellulose			Lignin		
	Temp [°C]	Exp [%]	Sim [%]	Diff [%p]	Exp [%]	Sim [%]	Diff [%p]	Exp [%]	Sim [%]	Diff [%p]	Exp [%]	Sim [%]	Diff [%p]
**30min**	**230**	91.36	88.06	3.30	40.99	41.00	-0.01	18.93	20.50	-1.57	31.41	33.67	-2.26
	**250**	90.65	86.78	3.87	38.75	40.98	-2.23	23.37	19.13	4.24	31.45	34.82	-3.37
	**270**	89.65	83.70	5.95	42.67	40.91	1.76	18.67	15.82	2.85	32.61	37.76	-5.15
	**290**	87.47	78.01	9.46	42.54	40.69	1.85	17.40	9.68	7.72	34.71	43.83	-9.12
**60min**	**230**	88.30	84.41	3.89	37.92	40.94	-3.02	17.91	16.65	1.26	35.21	37.03	-1.82
	**250**	86.35	78.13	8.22	39.16	40.76	-1.60	18.47	9.92	8.55	35.60	43.63	-8.03
	**270**	82.70	70.94	11.76	23.94	40.17	-16.23	18.78	2.34	16.44	51.30	52.41	-1.11
	**290**	72.52	68.26	4.26	23.17	38.26	-15.09	12.05	0.05	12.00	59.64	56.40	3.24
**R** ^**2**^		0.8193			0.6633			0.4724			0.8803		
**RMSE**		2.4683			2.8348			3.0029			1.7927		

Table 6 presents the simulation findings for herbaceous biomass, BE. The simulated mass yield varied between 64.18 and 81.73%. Nevertheless, the observed torrefaction results exhibited a variation of up to 10.66%, spanning from 55.83% to 90.24%. Difference between experimental and simulation data were detected for cellulose, with errors ranging from 1.56%p to 11.00%p. These errors were quite moderate when compared to WP results. Nevertheless, there were notable variations seen in the levels of hemicellulose (ranging from 1.77% to 20.26%) and lignin (ranging from 2.45% to 23.88%). The simulation results exhibited a tendency to overestimate the amount of lignin and underestimate the amount of hemicellulose. This discrepancy can be attributed to the greater predictive accuracy of Park et al. (2023)‘s equation for woody biomass [24]. Therefore, it is considered that adjustments to the kinetic parameters and additional corrections for cellulose, hemicellulose, and lignin should be necessary [44,45].

**Table 6:** Simulation result with previous kinetic parameters of BE.

		Mass			Cellulose			Hemicellulose			Lignin		
	Temp [°C]	Exp [%]	Sim [%]	Diff [%p]	Exp [%]	Sim [%]	Diff [%p]	Exp [%]	Sim [%]	Diff [%p]	Exp [%]	Sim [%]	Diff [%p]
**30min**	**230**	90.24	81.73	8.51	36.78	35.22	1.56	28.54	17.38	11.16	14.17	35.66	-21.49
	**250**	90.30	79.64	10.66	40.38	35.18	5.20	23.94	15.15	8.79	16.35	37.70	-21.35
	**270**	84.93	75.07	9.86	32.35	35.05	-2.70	30.47	10.21	20.26	19.53	42.54	-23.01
	**290**	77.72	68.80	8.92	40.39	34.62	5.77	16.06	3.57	12.49	26.05	49.93	-23.88
**60min**	**230**	84.85	76.53	8.32	46.12	35.12	11.00	15.84	11.87	3.97	20.77	40.89	-20.12
	**250**	76.64	69.51	7.13	42.48	34.81	7.67	6.98	4.34	2.64	29.62	48.99	-19.37
	**270**	67.47	65.40	2.07	40.38	33.70	6.68	3.54	0.24	3.30	37.88	54.36	-16.48
	**290**	55.83	64.18	-8.35	22.17	30.02	-7.85	1.77	0.00	1.77	54.66	57.11	-2.45
**R** ^**2**^		0.8653			0.6239			0.7340			0.8522		
**RMSE**		2.9499			2.3589			3.5466			6.9315		

### Simulation with modified kinetic parameters

**Fig 4:**
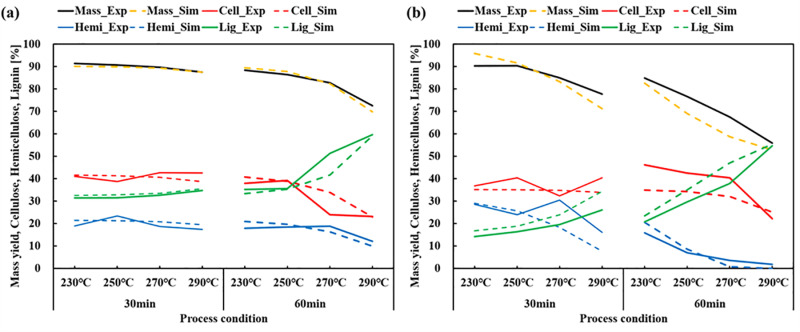
Comparison of experimental and simulation results (a) WP, (b) BE.

Kinetic parameters refer to the variables in the Arrhenius empirical equation that might vary based on the specific sample and conditions of the process [17,38,46]. The data presented in Tables 5 and 6 indicate that there was minimal degradation of cellulose compared to the experiment. Conversely, hemicellulose exhibited significant degradation. Moreover, there was a substantial disparity in the projected quantities of hemicellulose and lignin for herbaceous biomass. Based on previous study method, calibrations of kinetic parameters were conducted [47]. In order to address these issues, some kinetic parameters were adjusted and are outlined in Table 7. In order to calculate herbaceous biomass, weights of 1.2, 0.7, and 2.0 were ascribed to cellulose, hemicellulose, and lignin, respectively. The simulations for WP and BE were performed using the adjusted kinetic parameters, and the outcomes were displayed in Table 8 and Fig 4. The bulk yield varied between 90.09% and 72.52%. The inaccuracies were substantially diminished, ranging from 0.01% to 2.77%, in comparison to the prior model which had errors ranging from 3.30–11.76%p. There was a reduction in errors in cellulose, hemicellulose, and lignin. The RMSE and R^2^ metrics demonstrated superior performance as compared to the simulation results obtained with the previous kinetic settings.

**Table 7:** Modified multiple-step kinetic model for biomass.

Pyrolysis Reactions				Kinetic Parameters A(s^-1^), Ea(kcal/kmol)	
				Woody biomass	Herbaceous biomass
**Cellulose**					
1	Cell	→	CellA	1.5 × 10^14^ × exp(-45500/RT)	1.5 × 10^14^ × exp(-46000/RT)
2	CELLA	→	0.4C2H4O2 + 0.05C2H2O2 + 0.15CH3CHO + 0.2C6H6O3 + 0.35C2H5CHO + 0.15CH3OH + 0.3CH2O + 0.61CO + 0.36CO2 + 0.05H2 + 0.93H2O + 0.02HCOOH + 0.05C3H6O2 + 0.05G_CH4 + 0.2G_H2 + 0.61Char	2.5 × 10^6^ × exp(-18000/RT)	
3	CELLA	→	C6H10O5	1.8 × T × exp(-10000/RT)	
4	CELL	→	5H2O + 6Char	4 × 10^7^ × exp(-30500/RT)	4 × 10^7^ × exp(-30000/RT)
**Hemicellulose**					
5	HECELL	→	0.58HCE1+0.42HCE2	0.33 × 10^10^ × exp(-33000/RT)	0.33 × 10^10^ × exp(-30000/RT)
6	HCE1	→	0.6C5H8O4 + 0.2C3H6O2 + 0.12C2H2O2 + 0.2C5H4O2 + 0.4H2O + 0.08G_H2 + 0.16CO + 0.4H2O + 0.79CO2 + 0.05HCOOH + 0.69CO + 0.01G_CO + 0.01G_CO2 + 0.35G_H2 + 0.3CH2O + 0.9G_COH2 + 0.625G_CH4 + 0.375G_C2H4 + 0.875Char	3 × T × exp(-10618/RT)	
7	HCE1	→	0.4H2O + 0.79CO2 + 0.05HCOOH + 0.69CO + 0.01G{CO} + 0.01G{CO2} +0.35G{H2} + 0.3CH2O + 0.9 G{COH2} + 0.625 G{CH4} + 0.375 G{C2H4} + 0.875 CHAR	1.8 × 10^–3^ × T × exp(-2618/RT)	
8	HCE2	→	0.2H2O + 0.275CO + 0.175CO2 + 0.4CH2O + 0.1C2H5OH + 0.05C2H4O2 + 0.35CH3COOH + 0.025HCOOH + 0.25G_CH4 + 0.3G_CH3OH + 0.225G_C2H4 + 0.4G_CO2 + 0.725G_COH2 + Char	0.33 × 10^10^ × exp(-34000/RT)	

**Table 8:** Simulation result with modified kinetic parameters of WP and BE.

			Mass			Cellulose			Hemicellulose				Lignin		
		Temp [°C]	Exp [%]	Sim [%]	Diff [%p]	Exp [%]	Sim [%]	Diff [%p]	Exp [%]	Sim [%]	Diff [%p]	Exp [%]		Sim [%]	Diff [%p]
**WP**	**30** **min**	**230**	91.36	90.09	1.27	40.99	41.57	-0.58	18.93	21.46	-2.53	31.41		32.51	-1.10
		**250**	90.65	89.88	0.77	38.75	41.31	-2.56	23.37	21.28	2.09	31.45		32.76	-1.31
		**270**	89.65	89.28	0.37	42.67	40.59	2.08	18.67	20.78	-2.11	32.61		33.48	-0.87
		**290**	87.47	87.48	-0.01	42.54	38.69	3.85	17.40	19.50	-2.10	34.71		35.52	-0.81
	**60** **min**	**230**	88.30	89.43	-1.13	37.92	40.75	-2.83	17.91	20.94	-3.03	35.21		33.30	1.91
		**250**	86.35	87.72	-1.37	39.16	38.86	0.30	18.47	19.64	-1.17	35.60		35.29	0.31
		**270**	82.70	82.33	0.37	23.94	33.89	-9.95	18.78	16.31	2.47	51.30		41.62	9.68
		**290**	72.52	69.75	2.77	23.17	22.92	0.25	12.05	9.91	2.14	59.64		59.10	0.54
**R** ^**2**^			0.9742			0.7281			0.6432				0.8869		
**RMSE**			0.4560			1.4427			0.7990				1.2629		
**BE**	**30** **min**	**230**	90.24	95.78	-5.54	36.78	35.12	1.66	28.54	29.01	-0.47	14.17		16.75	-2.58
		**250**	90.30	91.56	-1.26	40.38	35.04	5.34	23.94	25.67	-1.73	16.35		18.77	-2.42
		**270**	84.93	83.22	1.71	32.35	34.78	-2.43	30.47	18.30	12.17	19.53		23.90	-4.37
		**290**	77.72	71.21	6.51	40.39	33.88	6.51	16.06	7.66	8.40	26.05		34.34	-8.29
	**60** **min**	**230**	84.85	82.54	2.31	46.12	34.92	11.20	15.84	20.51	-4.67	20.77		23.44	-2.67
		**250**	76.64	69.00	7.64	42.48	34.29	8.19	6.98	8.60	-1.62	29.62		34.98	-5.36
		**270**	67.47	58.71	8.76	40.38	32.05	8.33	3.54	0.77	2.77	37.88		46.98	-9.10
		**290**	55.83	52.84	2.99	22.17	25.08	-2.91	1.77	0.00	1.77	54.66		55.42	-0.76
**R** ^**2**^			0.9367			0.6169			0.7594				0.9538		
**RMSE**			1.8832			2.3383			2.0044				1.8523		

### Mass reduction model validation

**Fig 5:**
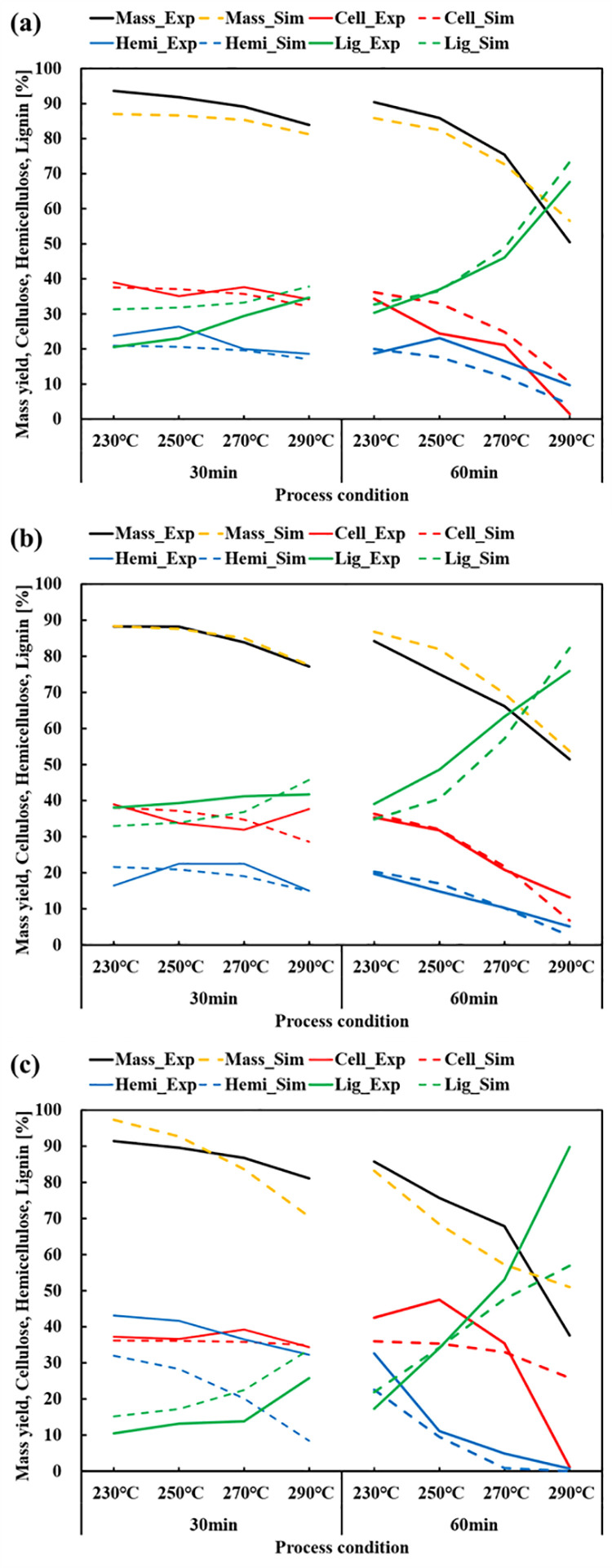
Comparison of experimental and simulation results (a) PP, (b) BB, (c) CC.

For the purpose of model validation, similar types of biomass were employed. For the purpose of validation, woody biomass was assessed using PP and BB, while herbaceous biomass was assessed using CC (Fig 5). The validation results were summarised in Table 9. The experimental mass yield for PP varied between 50.47% and 93.59%. The simulation results exhibited a comparable range, spanning from 56.55% to 87.04%. The cross-validated root mean square error (RMSE_cv_) was 1.6246, indicating the average difference between the predicted and actual values. R^2^_cv_ revealed a strong positive correlation, with a value of 0.9935. The prediction of cellulose, and lignin likewise exhibited excellent performance, with an R^2^ value of 0.91. But hemicellulose showed less performance compared with other parameters, showing 0.7849. BB also demonstrated a significant reliability. RMSE_cv_ for mass yield was lower, with a value of 1.0074, compared to PP. However, the R^2^ value was 0.9652, showing a less performance of the process. The prediction for cellulose and hemicellulose were similarly precise. The RMSE_cv_ for hemicellulose was 0.9101 and the R^2^cv was 0.8232, indicating that it performed better than PP. However, in the case of CC, the validation results were lower, which is in contrast to the earlier findings. The RMSE_cv_ for mass yield was 2.8463, while R^2^_cv_ was 0.7743. The predictions for cellulose, hemicellulose, and lignin showed a certain correlation with R^2^_cv_ values over 0.77. However, the RMSE_cv_ reached a high value of 2.8463, indicating worse performance.

**Table 9:** Simulation result of PP, BB and CC with modified kinetic parameters.

			Mass			Cellulose			Hemicellulose			Lignin		
		Temp [°C]	Exp [%]	Sim [%]	Diff [%p]	Exp [%]	Sim [%]	Diff [%p]	Exp [%]	Sim [%]	Diff [%p]	Exp [%]	Sim [%]	Diff [%p]
**PP**	**30** **min**	**230**	93.59	87.04	6.55	38.96	37.53	1.43	23.74	20.96	2.78	20.57	31.33	-10.76
		**250**	91.84	86.62	5.22	35.10	37.06	-1.96	26.37	20.61	5.76	23.06	31.81	-8.75
		**270**	89.13	85.33	3.80	37.63	35.72	1.91	20.00	19.60	0.40	29.40	33.27	-3.87
		**290**	83.93	81.25	2.68	34.17	32.13	2.04	18.63	17.07	1.56	34.62	37.78	-3.16
	**60** **min**	**230**	90.41	85.85	4.56	34.29	36.21	-1.92	18.70	20.02	-1.32	30.31	32.69	-2.38
		**250**	85.92	82.47	3.45	24.43	33.02	-8.59	23.12	17.64	5.48	36.95	36.54	0.41
		**270**	75.43	72.71	2.72	21.12	24.87	-3.75	16.54	12.08	4.46	46.05	48.80	-2.75
		**290**	50.47	56.55	-6.08	1.47	10.45	-8.98	9.71	4.23	5.48	67.66	73.34	-5.68
**R** ^**2**^			0.9935			0.9292			0.7849			0.9421		
**RMSE**			1.6246			1.7035			1.3991			2.028		
**BB**	**30** **min**	**230**	88.23	88.36	-0.13	38.93	38.06	0.87	16.46	21.58	-5.12	38.05	32.94	5.11
		**250**	88.17	87.56	0.61	33.75	37.17	-3.42	22.55	20.90	1.65	39.31	33.89	5.42
		**270**	83.85	84.99	-1.14	31.92	34.72	-2.80	22.52	19.05	3.47	41.23	36.82	4.41
		**290**	77.14	77.54	-0.40	37.65	28.56	9.09	15.03	14.86	0.17	41.70	45.69	-3.99
	**60** **min**	**230**	84.18	86.77	-2.59	35.34	36.32	-0.98	19.72	20.32	-0.60	39.05	34.81	4.24
		**250**	75.02	81.99	-6.97	31.75	32.01	-0.26	14.83	17.07	-2.24	48.57	40.44	8.13
		**270**	66.20	69.70	-3.50	20.84	21.75	-0.91	10.31	10.23	0.08	63.29	57.16	6.13
		**290**	51.45	53.69	-2.24	13.17	6.73	6.44	5.12	2.55	2.57	75.92	82.30	-6.38
**R** ^**2**^			0.9652			0.8312			0.8232			0.9260		
**RMSE**			1.0774			1.5116			0.9101			1.9893		
**CC**	**30** **min**	**230**	37.22	36.18	1.04	43.10	31.96	11.14	10.50	15.20	-4.70	37.22	36.18	1.04
		**250**	36.61	36.10	0.51	41.64	28.28	13.36	13.19	17.21	-4.02	36.61	36.10	0.51
		**270**	39.25	35.82	3.43	36.49	20.17	16.32	13.80	22.46	-8.66	39.25	35.82	3.43
		**290**	34.34	34.90	-0.56	32.24	8.44	23.80	25.79	33.64	-7.85	34.34	34.90	-0.56
	**60** **min**	**230**	85.76	83.19	2.57	42.52	35.97	6.55	32.65	22.60	10.05	17.28	21.89	-4.61
		**250**	75.69	68.41	7.28	47.49	35.32	12.17	11.11	9.48	1.63	34.07	34.23	-0.16
		**270**	67.84	57.24	10.60	35.41	33.02	2.39	4.91	0.84	4.07	53.05	47.62	5.43
		**290**	37.61	51.03	-13.42	1.12	25.83	-24.71	0.71	0.00	0.71	89.78	56.88	32.90
**R** ^**2**^			0.7743			0.9292			0.7849			0.9421		
**RMSE**			2.8463			1.7035			1.3991			2.0280		

### Mass reduction model verification

**Fig 6:**
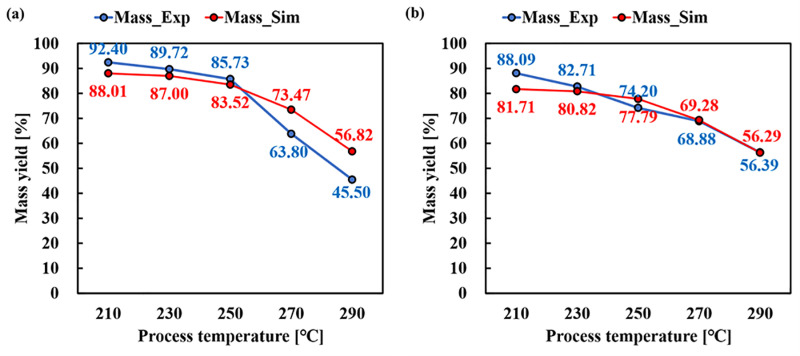
Comparison of experimental and simulation mass yield result (a) RC, (b) AP.

To verify the model, predictions were made based on the results of other study [48]. However, in the previous studies, only mass yield was considered, as the composition of chemical composition components was not known. The comparison results were expressed in Fig 6 and Table 10. For herbaceous biomass and woody biomass, rice husk (RC) and apple pruning branch pellet (AP) were selected. For RC, RMSE and R^2^ showed 3.3866% and 0.9222, respectively. AP showed a higher RMSE of 7.1165% compared to RC, but it had a high R^2^ of 0.9791, indicating a strong trend. The discrepancies observed in AP may be attributed to its higher lignin content, which affects decomposition rates differently than herbaceous biomass. Future modifications should consider further refining the kinetic parameters for lignin decomposition.

**Table 10:** Energy yield calculation of R^2^ and RMSE of biomass.

	RC	AP
**R** ^**2**^	0.9222	0.9791
**RMSE**	3.3866	7.1165

### Comparison of energy yield

**Fig 7:**
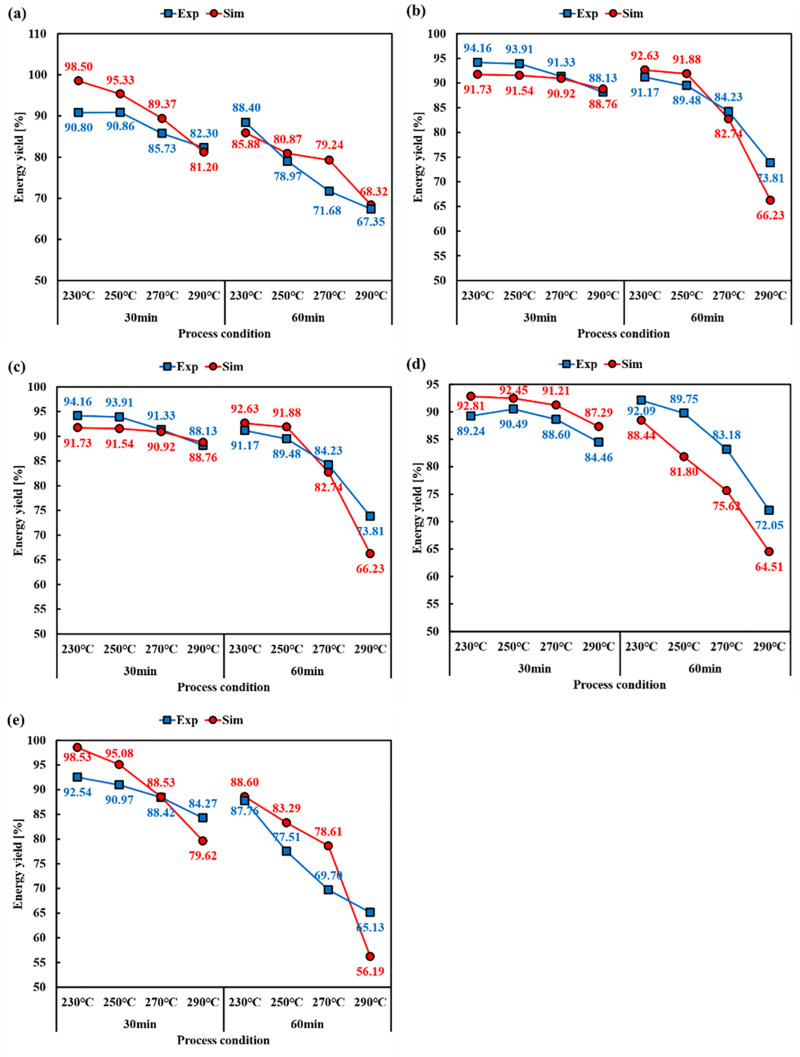
Energy yield comparison between prediction and experiment results (a) WP, (b) BE, (c) PP, (d) BB, and (e) CC.

Based on predicted values [49], calculating energy yields was attempted. For calorific value, the calorific value prediction model, was used. Energy yields of biomass, whose biomass were used for the prediction model, were depicted in Fig 7. As with the results of the mass loss model, WP showed high prediction accuracy. However, BE showed relatively low prediction accuracy. R^2^ and RMSE are summarised in Table 11. Despite the relatively low accuracy of BE, high accuracy was shown with a R^2^ of 0.8869 and a RMSE of 4.4962. In the case of WP, R^2^ was 0.9688 and RMSE was 1.1925, showing high accuracy. In the case of BB, the highest R^2^ was observed, showing 0.9656. However, the RMSE was 5.2656%. This was determined to be due to the low mass prediction rate at high temperatures. This trend also appeared in BB. The R^2^ and RMSE of CC were 0.7940 and 5.7879, respectively. The energy yield predictions at higher temperatures showed slightly larger deviations, suggesting that further calibration is required for biomass with lower lignin content.

**Table 11:** Energy yield calculation of R2 and RMSE of biomass.

	WP	BE	PP	BE	CC
**R** ^**2**^	0.9688	0.8869	0.9603	0.9656	0.7940
**RMSE**	1.1925	4.4962	3.1562	5.2656	5.7879

## Conclusions

Predicting changes in the composition of cellulose, hemicellulose, and lignin was the aim of this study in order to verify the mass reduction observed during thermochemical processes. To achieve this aim, the quantification of cellulose, hemicellulose, and lignin was performed by making use of previously published research results. Furthermore, the estimation of changes in cellulose, hemicellulose, and lignin concentrations as well as the quantification of mass loss were predicted through the application of kinetic parameters. However, the results obtained from applying kinetic parameters determined in previous research displayed discrepancies in comparison to the empirical data, thus requiring the modification of certain kinetic parameters. Subsequent to incorporating additional adjustments to accommodate herbaceous biomass as well as woody biomass, a comprehensive validation procedure was conducted. The RMSE_P_ values obtained for the components cellulose, hemicellulose, lignin, and mass yield in WP were 2.4683, 2.8348, 3.0029, and 1.7927, respectively. On the other hand, an RMSE_P_ range of 1.8523–2.3383 was observed for herbaceous biomass, BE. Following this, validation was performed utilising various biomass categories, including BB, PP, and CC, in an effort to predict mass yield using information gathered in prior investigations.

This study has established that it is feasible to forecast mass yield by considering the concentrations of lignin, cellulose, and hemicellulose. Further research should place emphasis on improving the accuracy of predicting herbaceous biomass, while simultaneously enhancing the precision of the model by integrating various lignocellulosic components.

## Supporting information

S1 File Source code for mass reduction prediction model. This file contains source code developed for the mass reduction prediction model during biomass torrefaction.
(DOCX)
