# Supplementary material for: Development and validation of mass reduction prediction model during torrefaction using biomass chemical composition analysis
Source: PLoS One. 2025 May 23;20(5):e0323940. doi: 10.1371/journal.pone.0323940 (PMC12101785; doi:10.1371/journal.pone.0323940)
Supplement: S1 File — This file contains source code developed for the mass reduction prediction model during biomass torrefaction. (DOCX) [file pone.0323940.s001.docx]

Development and Validation of Mass Reduction Prediction Model During Torrefaction Using Biomass Chemical Composition Analysis

Sunyong Park ^1†^, Kyeong Sik Kang ^2†^, Kwang Cheol Oh ^1^, Seok Jun Kim ^2^, Paudel Padam Prasad ^2,^ Seon Yeop Kim ^3^, Ha Eun Kim ^3^, Jae Youl Shin ^3^, DaeHyun Kim ^1,2,3*^

^1^ Agriculture and Life Science Research Institute, Kangwon National University, Hyoja 2 Dong 192-1, Chuncheon-si, Republic of Korea

^2^ Department of Interdisciplinary Program in Smart Agriculture, Kangwon National University, Hyoja 2 Dong 192-1, Chuncheon-si, Republic of Korea;

^3^ Department of Biosystems Engineering, Kangwon National University, Hyoja 2 Dong 192-1, Chuncheon-si, Republic of Korea;

**Supplement 1. code of mass reduction prediction model**

clear all

%%%%%%%%%%%%%%%%%%%

% Input data

MC1=10;

C=45.15;

H=6.12;

N=1.21;

O=47.52;

S=0.00;

%%%%%%%%%%%%%%%%%%%%%%%%%%%%%%%%%%%%%%%%%%%%%%%%%

HC=(H/C)*10; HC2=HC^2;

OC=(O/C); OC2=(O/C)^2;

VM=13.647-0.344*C+8.902*H+0.547*O-2.02*N+3.945*S;

FC=8.714+1.029*C-6.273*H+0.04*O+0.624*N-1.413*S;

VM2=VM^2;

FC2=FC^2;

FCVM=FC/VM;

FCVM2=(FC/VM)^2;

PA=VM+FC;

Ash1=100-PA;

Cell1=(-152.237+0.838*C-10.405*H+3.309*O-37.061*HC2+177.211*HC+20.086*OC2-136.126*OC+0.151*VM+0.391*FC-4.52*(FC/VM));

if Cell1<0

Cell=0;

end

Lig1=(-121.824+39.757*HC2-128.192*HC+23.719*OC2-56.877*OC-0.042*VM2+6.932*VM+0.000473*FC2-2.954*FC-45.172*FCVM2+239.132*FCVM);

if Lig1<0

Lig1=0;

end

Hemi1=(1465.909+0.212*C^2+0.66*H^2+0.101*O^2-33.374*C-49.249*H+10.983*O-66.583*HC2+363.635*HC+209.649*OC2-1329.208*OC-0.376*VM-0.673*FC);

if Hemi1<0

Hemi1=0;

end

TOTAL1=MC1+Cell1+Lig1+Hemi1+Ash1;

Cell=(100-Ash1)*Cell1/TOTAL1;

Lig2=(100-Ash1)*Lig1/TOTAL1;

Hemi=(100-Ash1)*Hemi1/TOTAL1;

MC=MC1;

Ash=Ash1;

TGL=0;

TANN=0;

TP=Cell+Lig2+Hemi+TGL+MC+TANN+Ash;

% Hardwood

LigC=0.17*Lig2; LigH=0.51*Lig2; LigO=0.32*Lig2;

% % Softwood

% LigC=0.342*Lig2; % LigH=0.569*Lig2; % LigO=0.089*Lig2;

% Grass

% LigC=0.376*Lig2; % LigH=0.394*Lig2; % LigO=0.229*Lig2;

SA_total=Cell+Hemi+LigC+LigH+LigO+Ash+MC;

%%%%%%%%%%%%%%%%%%%%%%%%%%%%%%%%%%%%%%%%%%%%%%%%%%%

Set_Temp=input('Set process temperature [C]:');

Set_min=60;

% Set_min=input('Set process time [min]:')

Set_Time=Set_min*60;

% Time progress

time=[1:1:Set_Time]';

% Number of nodes

N=55;

% Convert wood diameter from mm to m

Wood_L=75*1e-3;

% Convert steel thickness from mm to m

Steel_L=2*2*1e-3;

% Convert height from mm to m

hight=51*1e-3;

% Surrounding temperature

Tair=Set_Temp;

% Initial wood temperature

Ti=25;

% Steel properties

k_s=16.3; cp_s=502; den_s=7900; % SUS304 k=W/mK, cp=J/kgK

T_in=Ti+273.5;

Ts1=Tair+273.5;

% Node distance for wood and steel

Wood_dx=Wood_L/71;

Steel_dx=Steel_L/4;

WS_dx=Wood_dx/2+Steel_dx/2;

% Parameter used in differential equations

c_s=k_s/(den_s*cp_s*Steel_dx^2);

% Initial temperature conditions

Tz1=[Ts1 T_in T_in T_in T_in T_in T_in T_in T_in T_in T_in T_in T_in T_in T_in T_in T_in T_in T_in T_in T_in T_in T_in T_in T_in T_in T_in T_in T_in T_in T_in T_in T_in T_in T_in T_in T_in T_in T_in T_in T_in T_in T_in T_in T_in T_in T_in T_in T_in T_in T_in T_in T_in T_in Ts1];

% Solve ODE using Matlab solver

[t1,T1]=ode45('bark_mod1_odes',[0 Set_Time],Tz1',[ ],N,c_s,Ts1,Wood_dx);

for h=1:55

% Cellulose

k2(:,h) = (1.5e+14 .* exp(-45000 ./ (R * T_interp(:,h)))); % CELL -> CELLA

k3(:,h) = 2.5e+6 .* exp(-17000 ./ (R * T_interp(:,h)));

k4(:,h) = 1.8 * T_interp(:,h) .* exp(-10000 ./ (R * T_interp(:,h))); % CELLA -> C6H10O5

k5(:,h) = 4e+7 .* exp(-28000 ./ (R * T_interp(:,h))); % CELL -> 5*H2O + 6*CHAR

% Hemicellulose

k6(:,h) = 0.33e+10 .* exp(-32000 ./ (R * T_interp(:,h)));

k7(:,h) = 3 .* T_interp(:,h) .* exp(-10618 ./ (R * T_interp(:,h)));

k7_2(:,h) = 1.8e-3 .* T_interp(:,h) .* exp(-2618 ./ (R * T_interp(:,h)));

k8(:,h) = 0.33e+10 .* exp(-34000 ./ (R * T_interp(:,h)));

% Lignin

k9(:,h) = 1e+11 .* exp(-37200 ./ (R * T_interp(:,h)));

k10(:,h) = 6.7e+12 .* exp(-37500 ./ (R * T_interp(:,h)));

k11(:,h) = 3.3e+8 .* exp(-25500 ./ (R * T_interp(:,h)));

k12(:,h) = 1e+4 .* exp(-24800 ./ (R * T_interp(:,h)));

k13(:,h) = 1e+8 .* exp(-30000 ./ (R * T_interp(:,h)));

k14(:,h) = 4 * T_interp(:,h) .* exp(-12000 ./ (R * T_interp(:,h)));

k15(:,h) = 8.3e-2 * T_interp(:,h) .* exp(-8000 ./ (R * T_interp(:,h)));

k16(:,h) = 1e+7 .* exp(-24300 ./ (R * T_interp(:,h)));

% Metaplastic

k18(:,h) = 1e+6 .* exp(-24000 ./ (R * T_interp(:,h)));

k19(:,h) = 5e+12 .* exp(-50000 ./ (R * T_interp(:,h)));

k20(:,h) = 1.5e+12 .* exp(-71000 ./ (R * T_interp(:,h)));

k21(:,h) = 5e+11 .* exp(-75000 ./ (R * T_interp(:,h)));

k22(:,h) = 5e+12 .* exp(-71500 ./ (R * T_interp(:,h)));

k23(:,h) = 2e+12 .* exp(-50000 ./ (R * T_interp(:,h)));

k24(:,h) = 5e+12 .* exp(-71500 ./ (R * T_interp(:,h)));

% Extractives

k26(:,h) = 7e+12 .* exp(-45700 ./ (R * T_interp(:,h)));

k27(:,h) = 5e+1 .* exp(-11000 ./ (R * T_interp(:,h)));

k28(:,h) = 1.5e-2 .* exp(-6100 ./ (R * T_interp(:,h)));

% Evaporation

k29(:,h) = 1.0 * T_interp(:,h) .* exp(-8000 ./ (R * T_interp(:,h)));

end

function [Tdot]=bark_mod1_odes(t,T,flag,N,c_s,Ts,Wood_dx)

T(1)=Ts;

T(N)=Ts;

Tdot(1)=0;

Tdot(N)=0;

den_w=400;

k_s=16.3; cp_s=502; den_s=7900;

for m=1:55

cp_w(m)=1.3294*T(m)+674.3;

k_w(m)=0.13+3*10^-4*(T(m)-273.15);

c_w(m)=k_w(m)/(den_w*cp_w(m)*Wood_dx^2);

end
